# Supplementary material for: Evolution of VIM-1-Producing Klebsiella pneumoniae Isolates from a Hospital Outbreak Reveals the Genetic Bases of the Loss of the Urease-Positive Identification Character
Source: mSystems. 2021 Jun 1;6(3):e00244-21. doi: 10.1128/mSystems.00244-21 (PMC8269217; doi:10.1128/mSystems.00244-21)
Supplement: FIG S1 [file msystems.00244-21-sf001.pdf]

	Antibiotic	Kp_{VIM} 1	Kp_{VIM} 2	Kp_{VIM} 3	Kp_{VIM} 4	Kp_{VIM} 5	Kp_{VIM} 6	Kp_{VIM} 7	Kp_{VIM} 8	Kp_{VIM} 9	Kp_{VIM} 10	Kp_{VIM} 11	Kp_{VIM} 12	Kp_{VIM} 13	Kp_{VIM} 14	Kp_{VIM} 15	Kp_{VIM} 16	Kp_{VIM} 17	Kp_{VIM} 18
Aminoglycoside	Amikacin	16	16	17	16	16	16	17	18	17	16	16	16	17	29	15	25	27	25
	Gentamicin	7	8	8	8	8	8	9	9	7	9	7	8	8	11	8	9	10	14
	Kanamycin	7	7	7	7	7	7	8	8	7	7	7	7	7	15	7	14	12	23
	Streptomycin	7	7	7	7	7	7	7	7	7	7	7	7	7	7	7	7	7	14
	Sulfonamides	7	7	7	7	7	7	7	7	7	7	7	7	7	7	7	7	7	7
Quinolone	Ciprofloxacin	25	26	26	26	27	26	19	19	25	26	26	13	13	28	25	26	25	36
	Nalidixic acid	18	19	19	19	19	20	11	9	19	19	19	7	7	17	19	19	22	27
Trimethoprim	Trimethoprim	7	7	7	7	7	7	7	7	7	7	7	7	7	7	7	7	7	18
Phenicol	Chloramphenicol	7	7	7	7	7	7	7	7	7	7	7	7	7	7	7	7	7	24
Tetracycline	Tigecycline	22	23	24	23	23	24	17	17	22	22	24	19	19	18	23	23	25	23
	Tetracycline	7	7	7	7	7	7	7	7	7	7	7	7	7	7	7	21	21	23
Macrolide	Erythromycin	8	8	8	8	8	8	7	7	8	8	8	10	11	8	8	8	7	9
	Azithromycin	12	12	14	16	17	14	14	16	15	14	15	19	18	17	15	16	15	17
Carbapenem	Imipenem	23	25	25	25	24	25	22	23	22	23	23	22	22	23	22	23	7	21
	Doripenem	17	18	18	17	17	17	16	15	18	18	18	16	16	17	18	18	7	18
	Ertapenem	21	21	22	21	21	21	19	18	20	21	22	19	19	19	22	21	7	22
	Meropenem	20	20	20	19	20	20	19	18	19	20	20	19	18	19	20	21	7	18
Other ß-lactam	Amoxicillin	7	7	7	7	7	7	7	7	7	7	7	7	7	7	7	7	7	7
	Ticarcillin	7	7	7	7	7	7	7	7	7	7	7	7	7	7	7	7	7	7
	Piperacillin	7	7	7	7	7	7	7	7	7	7	7	7	7	7	7	7	7	8
	Cefepime	17	17	18	19	16	16	14	13	15	16	16	13	13	13	16	17	7	19
	Ceftaroline	7	7	7	7	7	7	7	7	7	7	7	7	7	7	7	7	7	7
	Amoxicillin-clavulanate	11	10	10	10	7	7	7	7	7	7	7	7	7	7	7	7	7	7
	Ceftazidime	7	7	7	7	7	7	7	7	7	7	7	7	7	7	7	7	7	7
	Piperacillin-tazobactam	9	9	9	9	7	7	7	7	7	7	7	8	8	7	7	7	7	7
	Cefoxitin	11	12	11	11	10	10	7	7	9	10	10	7	7	7	10	10	7	12
	Cefuroxime	7	7	7	7	7	7	7	7	7	7	7	7	7	7	7	7	7	7
	Ticarcillin-clavulanate	10	9	10	10	10	10	12	12	10	10	10	11	11	11	10	10	9	7
	Aztreonam	10	10	10	10	8	8	8	9	7	8	7	7	7	7	8	10	9	37
	Mecillinam	7	7	7	7	7	7	7	7	7	7	7	7	7	7	7	7	7	7
	Moxalactam	9	9	9	9	9	8	7	7	8	9	9	7	7	7	9	10	7	11
	Cefotaxime	8	9	8	8	8	7	7	7	7	7	7	7	7	7	7	7	7	8
	Ceftriaxone	16	16	16	16	16	16	15	15	15	16	15	14	15	13	16	16	11	18
Polymyxine	Colistin MIC (µg/ml)	2	2	2	2	2	2	2	2	2	2	2	2	2	2	2	2	64	2

Fig S1 *Antibiotic susceptibility of the 18 VIM-1 isolates.* Antibiotic susceptibility was determined by antibiotic disk diffusion, except for Colistin, the MIC of which was determined by microdilution. Numbers indicate diameter of the inhibition zones (MIC for colistin). Susceptible, Intermediate and Resistant (SIR) categories were determined according to CLSI guidelines and indicated in green, yellow and red respectively. In purple are indicated isolates for which a further reduction in susceptibility to cefepime and cefoxitin was observed compared to KP_{VIM}1.
