# Supplementary material for: Evolution of VIM-1-Producing Klebsiella pneumoniae Isolates from a Hospital Outbreak Reveals the Genetic Bases of the Loss of the Urease-Positive Identification Character
Source: mSystems. 2021 Jun 1;6(3):e00244-21. doi: 10.1128/mSystems.00244-21 (PMC8269217; doi:10.1128/mSystems.00244-21)
Supplement: TABLE S1 [file msystems.00244-21-st001.pdf]

Table S1: Isolates from the outbreak characterized in this work

**Kp isolates**	**Date of isolation**	**Source of isolation**	**Biosample**
$KP_{VIM}1$	06 Oct 2005	Urine	SAMN14419408
$KP_{VIM}2$	21 Nov 2005	Throat swab	SAMEA7707542
$KP_{VIM}3$	01 Dec 2005	Urine	SAMEA7707543
$KP_{VIM}4$	28 Nov 2005	Rectal swab	SAMEA7707544
$KP_{VIM}5$	28 Nov 2005	Blood	SAMEA7707545
$KP_{VIM}6$	12 Dec 2005	Blood	SAMEA7707546
$KP_{VIM}7$	09 Dec 2016	Rectal swab	SAMEA7707547
$KP_{VIM}8$	06 Jan 2006	Urine	SAMEA7707548
$KP_{VIM}9$	10 Feb 2006	Blood	SAMEA7707549
$KP_{VIM}10$	27 Feb 2006	Urine	SAMEA7707550
$KP_{VIM}11$	16 Mar 2006	Sputum	SAMEA7707551
$KP_{VIM}12$	03 Apr 2006	Catheter	SAMEA7707552
$KP_{VIM}13$	31 Ma r2006	Blood	SAMEA7707553
$KP_{VIM}14$	03 May 2006	Throat swab	SAMEA7707554
$KP_{VIM}15$	27 Oct 2006	Bile	SAMEA7707555
$KP_{VIM}16$	24 May 2007	Urine	SAMEA7707556
$KP_{VIM}17$	03 Jul 2007	Rectal swab	SAMEA7707557
$KP_{VIM}18$	06 Sep 2007	Urine	SAMEA7707558
