# Supplementary material for: Evolution of VIM-1-Producing Klebsiella pneumoniae Isolates from a Hospital Outbreak Reveals the Genetic Bases of the Loss of the Urease-Positive Identification Character
Source: mSystems. 2021 Jun 1;6(3):e00244-21. doi: 10.1128/mSystems.00244-21 (PMC8269217; doi:10.1128/mSystems.00244-21)
Supplement: TABLE S2 [file msystems.00244-21-st002.pdf]

Table S2: KP$_{VIM}$ chromosome, plasmids and antibiotic resistance genes

Replicon		Chromosome	pKP1-1	pKP1-2	pKP1-3	pKP1-4	pKP3-5	pKP3-6	pKP3-7
Isolates			all isolates	all isolates	all isolates	KP$_{VIM}$1, KP$_{VIM}$11, KP$_{VIM}$16, KP$_{VIM}$17	KP$_{VIM}$3-8	KP$_{VIM}$3-6	KP$_{VIM}$3-8
replicon			IncFIB/IncFII	IncR/ IncFIA	IncL/M	IncFII	Linear	HCN#	HCN#
size (base pairs)		5351626	227556	110924	76065	80027	34017	2811	3861
ARG	Aminoglycoside		*aac(3)-IIa*	*aac(6')-Ib*<br>*aph(6)-Id*<br>*aph(3'')-Ib*	*aacA4*<br>*aadA1*(4)				
	ß-lactam	*bla*$_{SHV-11}$	*bla*$_{SCO-1}$<br>*bla*$_{TEM-1B}$	*bla*$_{TEM-1A}$(2)<br>*bla*$_{OXA-9}$(1)<br>*bla*$_{SHV-12}$	*bla*$_{VIM-1}$				
	Fluoroquinolone	*oqxA*<br>*oqxB*		*aac(6')Ib-cr*<br>*QnrA1*(1)					
	Fosfomycin	*fosA*							
	Macrolide				**msr*(E)(1)<br>**mph*(E)(1)				
	Phenicol			*catA2*(3)	*catA1*<br>*catB2*				
	Sulfonamide			*sul2*<br>*sul1*	*sul1*				
	Tetracycline			*tet*(D)					
	Trimethoprim			*dfrA14*	*dfrB1*				

High copy number plasmid; * present in two copies; (1) missing in KP$_{vim}$14; (2) missing in KP$_{vim}$4; (3) missing in KP$_{vim}$3, 5, 6 and 8; (4) missing in KP$_{vim}$5, 9, 11, 12 and 13
