# Supplementary material for: Evolution of VIM-1-Producing Klebsiella pneumoniae Isolates from a Hospital Outbreak Reveals the Genetic Bases of the Loss of the Urease-Positive Identification Character
Source: mSystems. 2021 Jun 1;6(3):e00244-21. doi: 10.1128/mSystems.00244-21 (PMC8269217; doi:10.1128/mSystems.00244-21)
Supplement: TABLE S3 [file msystems.00244-21-st003.pdf]

**Table S3**: [#]*K. pneumoniae* Isolates sharing similar IncL/M *bla*$_{VIM-1}$ plasmids

| Accession | Name | Year | ST | wzi | Carbapenemase |
|---|---|---|---|---|---|
| GCA_900516585.1 | EuSCAPE_ES080 | 2013 | ST15 | *wzi24* | VIM-1 |
| GCA_900517305.1 | EuSCAPE_ES221 | 2014 | ST483 | *wzi346* | VIM-1 |
| GCA_900517325.1 | EuSCAPE_ES220 | 2014 | ST483 | *wzi346* | VIM-1 |
| GCA_900501155.1 | EuSCAPE_ES133 | 2014 | ST15 | *wzi24* | VIM-1 |
| GCA_002903925.1 | LBMM1302 | 2016 | ST11 | *wzi24* | VIM-1; OXA-48 |
| GCA_008868035.1 | A03/17 | 2012 | ST15 | *wzi24* | VIM-1 |
| GCA_008868075.1 | B04/04 | 2012 | ST11 | *wzi24* | VIM-1 |
| GCA_009746265.1 | ST340-VIM-1 | 2013-2014 | ST340 | *Wzi50* | VIM-1 |
| GCA_009746285.1 | 8-ST11-VIM-1 | 2013-2014 | ST11 | *wzi24* | VIM-1 |
| GCA_009746405.1 | 2-ST15-VIM-1 | 2013-2014 | ST15 | *wzi24* | VIM-1 |

[#]As identified at the NCBI, July 2020
