# Supplementary material for: Evolution of VIM-1-Producing Klebsiella pneumoniae Isolates from a Hospital Outbreak Reveals the Genetic Bases of the Loss of the Urease-Positive Identification Character
Source: mSystems. 2021 Jun 1;6(3):e00244-21. doi: 10.1128/mSystems.00244-21 (PMC8269217; doi:10.1128/mSystems.00244-21)
Supplement: FIG S2 [file msystems.00244-21-sf002.pdf]

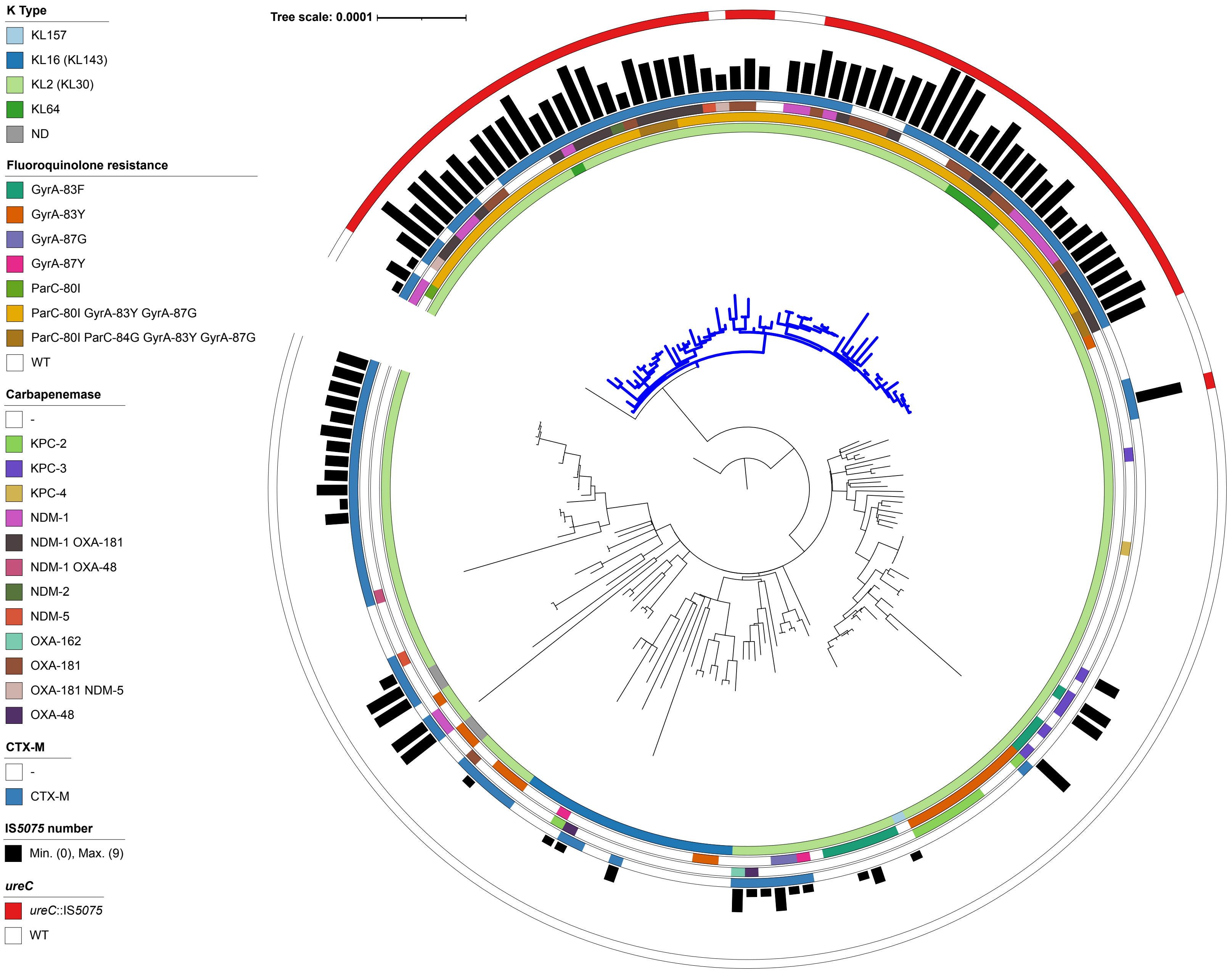

Fig. S2: Core genome phylogeny of *K. pneumoniae* ST14 isolates. Phylogeny was obtained by using Parsnp (Treangen TJ, Ondov BD, Koren S, Phillippy AM. 2014. Genome Biol 15:524 https://doi.org/10.1186/preaccept-2573980311437212) considering 174 genomes passing our quality threshold. K-type, mutations in *gyrA* and *parC* QRDR, carbapenemase genes, *bla*$_{\text{CTX-M}}$ genes, copy-number of IS*5075* and related ISs and IS insertion in *ureC* are shown on circles from inside to outside as indicated in the figure key (left). The *ureC* deficient lineage is in blue. Tree was mid-point rooted.
