# Supplementary material for: Evolution of VIM-1-Producing Klebsiella pneumoniae Isolates from a Hospital Outbreak Reveals the Genetic Bases of the Loss of the Urease-Positive Identification Character
Source: mSystems. 2021 Jun 1;6(3):e00244-21. doi: 10.1128/mSystems.00244-21 (PMC8269217; doi:10.1128/mSystems.00244-21)
Supplement: FIG S3 [file msystems.00244-21-sf003.pdf]

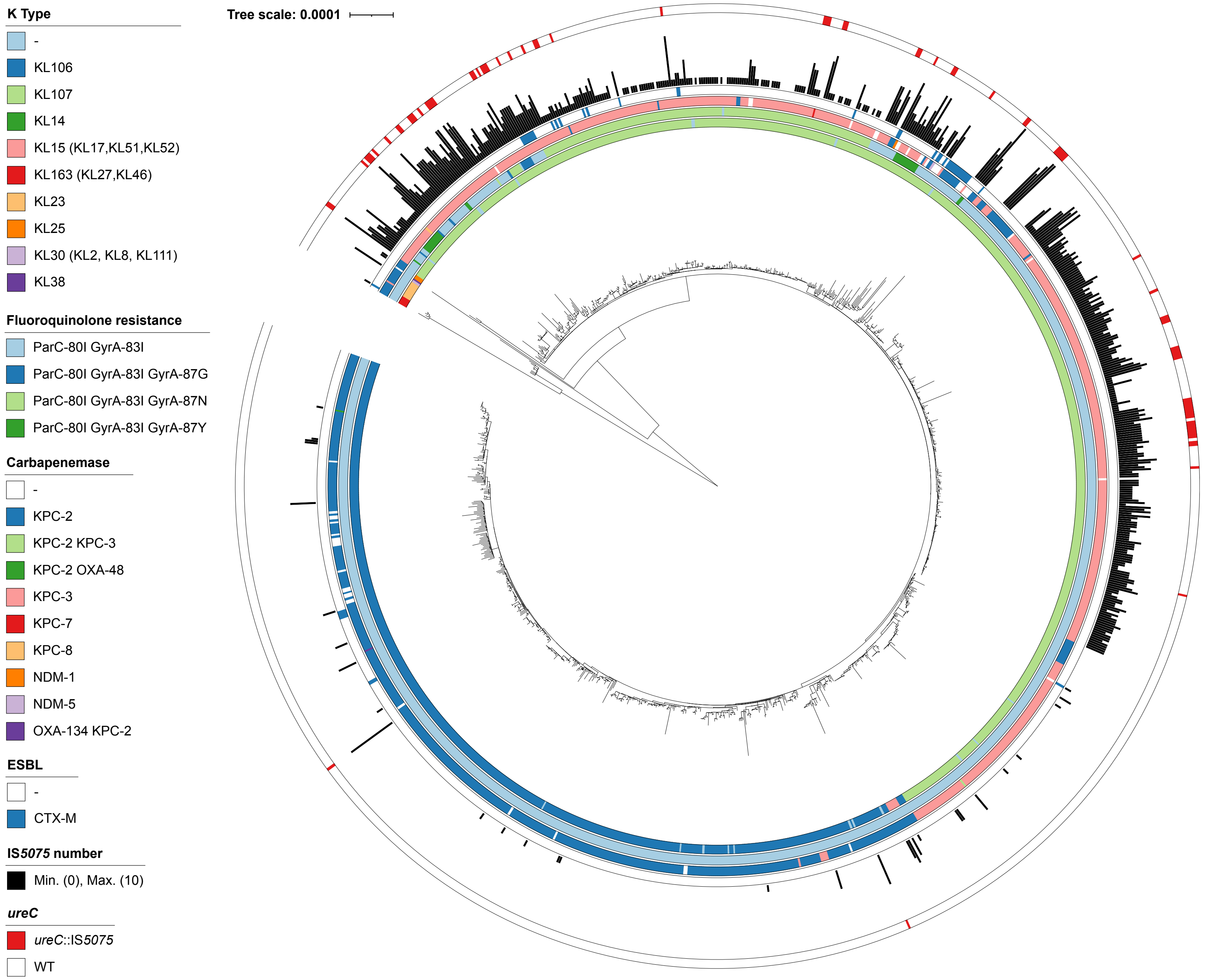

Fig. S3: *Core genome phylogeny* of K. pneumoniae *ST258 isolates*. Phylogeny was obtained by using Parsnp (Treangen TJ, Ondov BD, Koren S, Phillippy AM. 2014. Genome Biol 15:524 https://doi.org/10.1186/preaccept-2573980311437212).considering 1155 genomes passing the quality threshold. K-type, mutations in *gyrA* and *parC* QRDR, carbapenemase genes, *bla*$_{\text{CTX-M}}$ genes, copy-number of IS*5075* and related ISs and IS insertion in *ureC* are annotated by circles from inside to outside as indicated in the figure key (left).
