# Supplementary material for: Evolution of VIM-1-Producing Klebsiella pneumoniae Isolates from a Hospital Outbreak Reveals the Genetic Bases of the Loss of the Urease-Positive Identification Character
Source: mSystems. 2021 Jun 1;6(3):e00244-21. doi: 10.1128/mSystems.00244-21 (PMC8269217; doi:10.1128/mSystems.00244-21)
Supplement: FIG S4 [file msystems.00244-21-sf004.pdf]

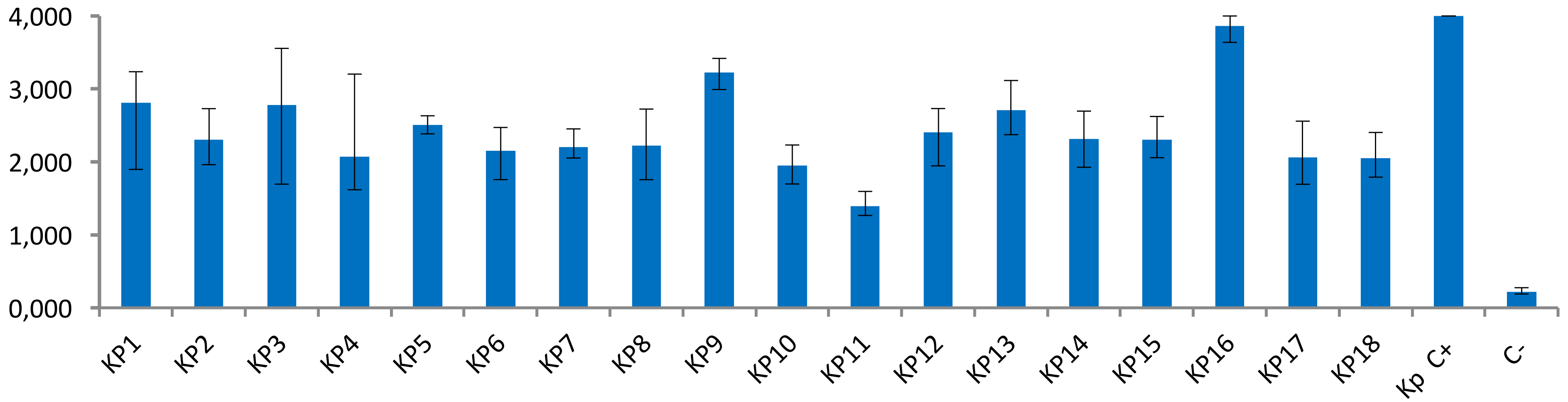

Fig. S4: *Biofilm formation by ST39 isolates.* Biofilm formation of the 17 ST39 isolates was quantified by using crystal violet staining. Absorbance values (n = 4, mean ± standard error of the mean) are indicated. *K. pneumoniae* strain LM21 was used as a positive control (Kp C+), no bacteria were added in the negative control (C-).
